# P01-18 Physical activity for children and youth with Physical disabilities - a case study on implementation in the municipality-setting

**DOI:** 10.1093/eurpub/ckac095.018

**Published:** 2022-08-29

**Authors:** Charlotte Præst, Thomas Skovgaard

**Affiliations:** Department of Sports Science and Clinical Biomechanics: Active Living, University of Southern Denmark, Odense, Denmark

**Keywords:** Physical activity, Children and youth, Physical disabilities, Municipal practices, Implementation

## Abstract

**Background:**

The Convention on the Rights of Persons with Disabilities by the United Nations states that physical activity (PA) is a human right for children and youth with disabilities, and they must experience equal opportunities for this. Nonetheless, compared to the population at large, this group participate less in PA, being 16-62% less likely to meet PA-guidelines.

While previous studies, mainly based on identifying facilitators and barriers for PA-participation, showed that promoting PA for children and youth with disabilities is a complex task, requiring multidisciplinary approaches targeting several levels in the individual's life, not much is known about how this is actually handled and realised in. Therefore, this study aimed to assess how practices in local settings influence PA-implementation for children and youth with physical disabilities.

**Methods:**

The study was designed as a qualitative multiple case study with two danish municipalities. A total of 23 semi-structured interviews with stakeholders from different municipal departments were conducted and local policy documents were included. Thematic analysis was performed, based on Winter's integrated implementation model, Lipsky's theory on street-level bureaucrats, and Gittell's theory on relational coordination.

**Results:**

PA-implementation for children and youth with physical disabilities is a complex challenge, involving many stakeholders and departments. The study found that efficient and sustainable performance can be improved by strengthening intersectoral collaboration, identifying shared goals and ambitions regarding PA for children and youth and, more concretely, supporting the work done by relevant local governmental departments and the schooling system. One of the municipalities had an explicit political focus on parasport and employed a parasport consultant, which had a positive influence on PA promotion across departments. In both municipalities consultants, coordinating cross-sectional processes, seemed to play an essential role. Additionally, the findings supported Michael Lipsky's theory regarding how street-level bureaucratic behaviour influence implementation processes and performances.

**Conclusions:**

The organisational structure of municipalities challenges performances on PA-implementation for children and youth with disabilities. Hopefully, the findings can inspire and support various stakeholders to strengthen their efforts to install high-performance and collaboration in order to establish quality, sustainable and diversified possibilities for PA participation among children and youth with disabilities.

